# 2275: Essential amino acid supplementation improves lipid metabolism in older adults with elevated triglycerides

**DOI:** 10.1017/cts.2017.33

**Published:** 2018-05-10

**Authors:** Bryce J. Marquis, Eugenia Carvahlo, Nicholas Hurren, Robert R. Wolfe, Elisabet Borsheim

## Abstract

OBJECTIVES/SPECIFIC AIMS: This study will assess the effect of essential amino acid (EAA) supplementation on plasma triglyceride (TG) in elderly adults. We will also explore the mechanisms mediating EAA mediated changes in fat metabolism and to suggest promising routes to refine therapy of hypertriglyceridemia. METHODS/STUDY POPULATION: In total, 7 nondiabetic male and female subjects ages 50–75 years with elevated plasma TG levels (130–500 mg/dL) were recruited to participate in an acute (5 h) and long-term (8 wk) EAA supplementation study. We measured changes in regional and whole body fat metabolism, including changes in body composition, plasma TG levels, whole body fat metabolic rates, tissue mitochondrial respiratory capacity, and metabolomic profiles before and after supplementation. RESULTS/ANTICIPATED RESULTS: Long-term EAA supplementation decreased fasted plasma TG levels by 19% (*p*<0.01). Metabolomics of skeletal muscle found acute EAA supplementation resulted in increased EAA metabolic products while long-term supplementation resulted in increased anaplerosis [flux into the tricarboxylic acid cycle (TCA) intermediate pool] and anaplerotic substrates [propionyl (*p*<0.01) and succinyl (*p*<0.01) carnitine] and intermediates of long-chain fatty acid metabolism [stearoyl (*p*<0.01) and myristoyl (*p*<0.05) carnitine]. However, tissue level respiratory capacity appeared to be unaffected by EAA supplementation. DISCUSSION/SIGNIFICANCE OF IMPACT: EAA supplementation has potential to improve lipid metabolism and plasma TG levels in non-diabetic older adults. Mitochondrial metabolomics suggest that insufficient TCA pool size may limit tissue fatty acid oxidation and may provide an additional route for nutritional therapy.

